# Genetic Polymorphism of SUMO-Specific Cysteine Proteases − SENP1 and SENP2 in Breast Cancer

**DOI:** 10.1007/s12253-016-0064-7

**Published:** 2016-05-13

**Authors:** Alicja Mirecka, Zbigniew Morawiec, Katarzyna Wozniak

**Affiliations:** 1 Department of Molecular Genetics, Faculty of Biology and Environmental Protection, University of Lodz, Lodz, Poland; 2 Department of Surgical Oncology, N. Copernicus Hospital, Lodz, Poland

**Keywords:** Breast cancer, SENP1 gene, SENP2 gene, Gene polymorphism, SUMO modification

## Abstract

SENP proteases take part in post-translational modification of proteins known as sumoylation. They catalyze three distinct processes during sumoylation: processing of SUMO protein, deconjugation of SUMO from the target protein, and chain editing which mentions to the dismantling of SUMO chain. Many proteins that are involved in the basic processes of cells, such as regulation of transcription, DNA repair or cell cycle control, are sumoylated. The aim of these studies was to investigate an association between polymorphic variants (SNPs) of the *SENP1* gene (c.1691 + 36C > T, rs12297820) and *SENP2* gene (c.902C > A, p.Thr301Lys, rs6762208) and a risk of breast cancer occurrence. We performed a case-control study in 324 breast cancer cases and 335 controls using PCR-RLFP. In the case of the *SENP1* gene polymorphism we did not find any association between this polymorphism and breast cancer risk. In the case of *SENP2* gene polymorphism we observed higher risk of breast cancer for carriers of the A allele (OR =1.33; 95 % CI 1.04–1.69). Our analysis also showed the genotype C/C (OR =0.67, 95 % CI 0.48–0.93) and the allele C (OR =0.75, 95 % CI 0.59–0.69) of this polymorphism decrease a risk of breast cancer. We also checked the distribution of genotypes and frequency of alleles of the *SENP1* and *SENP2* genes polymorphisms in groups of patients with different hormone receptor status, patients with positive and negative lymph node status and patients with different tumor grade. Odds ratio analysis showed a higher risk of metastases in women with the genotype C/C (OR =2.07, 95 % CI 1.06–4.05) and allele C (OR =2.10 95 % CI 1.10–4.01) of the c.1691 + 36C > T *SENP1* gene polymorphism. Moreover, we observed reduced risk in women with the allele T (OR =0.48, 95 % CI 0.25–0.91) in this polymorphic site. In the case of *SENP2* gene polymorphism we observed that the A/A genotype correlated with the lack of estrogen receptor (OR =1.94, 95 % CI 1.04–3.62). Our results suggest that the variability of the *SENP1* and *SENP2* genes may play a role in breast cancer occurrence. Further studies are needed to clarify their biological functions in breast cancer.

## Introduction

Small ubiquitin-like modifiers (SUMO) conjugation to proteins is a reversible post-translational modification, which may affect the function, sub-cellular localization and expression of proteins. Proteins, which are sumoylated take part in many cellular processes including regulation of transcription, DNA repair, nuclear transport and cell cycle control. Mammals express four different SUMO isoforms: SUMO-1, SUMO-2, SUMO-3 and SUMO-4. However, post-translational modification of proteins by SUMO-4 is questionable, as it remains in the inactive form in vivo [1, 2]. SUMO-2 and SUMO-3 share 95-97 % sequence homology and hence are collectively referred to as SUMO-2/3. SUMO-2/3 efficiently forms polymeric chains while in contrast SUMO-1 creates polymeric chains rarely. SUMO modification is a highly dynamic process, catalyzed by SUMO-specific activating (E1), conjugating (E2) and ligating (E3) enzymes. Sumoylation of target proteins results in the formation of isopeptide (amide) bond between the C-terminal glycine of SUMO and the ε-amino group of lysine within the target proteins [2].

During SUMO metabolism SUMO-specific proteases (SENPs) catalyze three distinct processes: processing, deconjugation and chain editing. SUMO proteins are expressed as precursor proteins that carry a C-terminal extension of variable length (2–11 amino acids) found after a conserved di-glycine motif. To function as a modifier of target proteins, the C-terminal di-glycine motif of the SUMO proteins must be exposed by the action of SUMO specific protease. SUMO processing activity of SENPs is responsible for cleavage after the C-terminal di-glycine motif. SUMO deconjugation activity of SENPs is required for the cleavage of amide bond between the C-terminus of the mature SUMO and the ε-amino group of the target lysine within the substrates. Chain editing refers to the dismantling of SUMO chain [3].

Six SUMO-specific SENPs have been identified in humans, SENPs 1, 2, 3, 5, 6 and 7. There are categorized into three independent subfamilies: SENP1 and SENP2, SENP3 and SENP5, and SENP6 and SENP7 proteases as the third subfamily. Unlike endopeptidase activity, all SENP family members exhibit isopeptidase activity to cleave the isopeptide bond between the glycine residue of SUMO and the lysine side chain of the substrate. The catalytic activity is maintained within a conserved 200 amino acid region in the C-terminal domain consisting of several highly conserved amino acids (cysteine, histidine and aspartic acid). SENPs possess a large N-terminal domain with minimal or no homology to each other’s domain. It has been suggested that the diversified N-terminal domains of SENP proteases determine their substrate specificity by controlling their sub-cellular localization [4]. It was shown that SENP1 localizes to the nucleoplasm, SENP2 localizes to the nuclear side of the nuclear pore complex (NPC), SENP3 and SENP5 localize to the nucleolus, and SENP6 localizes predominantly to the cytoplasm [4, 5]. Studies also indicate that the non-covalent binding of SUMO proteases to SUMO protein through salt bridge is essential for theirs enzymatic activities - to hydrolyze the C-terminal region of SUMO (hydrolase activity) and to remove SUMO from SUMO-conjugated substrates (isopeptidase activity) [6]. The 6 mammalian SENP enzymes can deconjugate mono-sumoylated proteins or dissemble polymeric SUMO side chains. SENP1 and SENP2 can desumoylate cellular substrates that are modified by any of the 3 SUMO isoforms while the remaining 4 SENPs are more efficient at deconjugating SUMO-2/3 than SUMO-1 [2].

The expression of several SENPs is altered in numerous cancers [7]. An elevated SENP1 level was observed in thyroid oncocytic adenocarcinoma [8], prostate cancer [9–11] and pancreatic ductal adenocarcinoma (PDAC) [12]. Moreover, it was shown that SENP1 expression directly correlates with prostate cancer aggressiveness and recurrence [13]. SENP1 was also over expressed in most of colon cancer tissues. Results of experiments with siRNA to inhibit SENP1 expression in DLD-1 colon cancer cell line, suggest a potential role for SENP1 in colon cancer cell proliferation, tumor formation and cell cycle progression [14]. SENP2 has been reported to play a critical role in the control of hepatocellular carcinoma (HCC) cell growth by modulating the stability of β-catenin [15]. Moreover, SENP2 functions as a tumor metastasis suppressor in bladder cancer. The effects of SENP2 on bladder cancer invasion are partially mediated by inhibiting the expression of MMP13 [16].

According to our knowledge the gene polymorphism encoding SENP proteases has not been investigated so far in terms of assessment of breast cancer risk. This fact prompted us to investigate the correlation between polymorphic variants (SNPs) of the *SENP1* gene (c.1691 + 36C > T, rs12297820) and *SENP2* gene (c.902C > A, p.Thr301Lys, rs6762208) and breast cancer risk. We also studied an association between the polymorphisms of the *SENP1* and *SENP2* genes and clinical characteristics of breast cancer patients such as lymph node status, tumor grade, hormone receptors (estrogen and progesterone receptors) and epidermal growth factor receptor (HER2) expression.

## Materials and Methods

### Patients

Blood samples were obtained from 324 women (mean age 60 years) with sporadic breast cancer treated at the Department of Surgical Oncology, N. Copernicus Hospital (Lodz, Poland). The clinical characteristic of breast cancer patients is presented in Table 1. Blood was collected before surgical treatment and chemotherapy. The control group (335 women) consisted of age-matched women who were not diagnosed with cancer and recruited from Commune Health Clinic in Rzgow and Institute Polish Mother’s Health Center (Lodz, Poland). The Local Ethic Committee approved the study and each patient gave a written consent.

**Table 1:** The clinical characteristics of breast cancer patients

Characteristic	Number of patients
Age	
Range: 32–92	324
Mean age ± SD: 60 ± 11	
*Carcinoma ductale*	248
*Carcinoma intraductale*	13
*Carcinoma lobulare*	37
Other	26
Tumor grade by Bloom-Richardson grading system	
1	19
2	101
3	117
Not determined	87
Metastases in lymph nodes	
positive	102
negative	133
Not determined	89
ER	
positive	235
negative	88
Not determined	1
PR	
positive	205
negative	118
Not determined	1

### Genomic DNA Isolation

Genomic DNA was prepared from peripheral blood of breast cancer patients and healthy individuals by using of commercial AxyPrep™ Blood Genomic DNA Miniprep Kit (Axygen Biosciences, CA, USA), as recommended by the manufacturer.

### Selection of Polymorphism and Primers Design

We obtained a list of SNPs in the *SENP1* and *SENP2* genes from the public domain of the National Center for Biotechnology Information the Single Nucleotide Polymorphisms database (NCBI dbSNP) at http://www.ncbi.nlm.nih.gov/snp. For this study we chose the c.1691 + 36C > T (rs12297820) polymorphism of the *SENP1* gene and the c.902C > A, p.Thr301Lys (rs6762208) polymorphism of the *SENP2* gene. Primers were designed according to the published nucleotide sequence in ENSEMBL database and using Primer3Plus software.

### Genotype Determination

The restriction fragment length polymorphism reaction (PCR-RFLP) was used to determine the genotypes of the c.1691 + 36C > T and the c.902C > A polymorphisms of the *SENP1* and *SENP2* genes, respectively. PCR reaction was performed in a total reaction volume of 25 μl containing 50 ng of genomic DNA, 1 U Biotools DNA polymerase (Biotools, Madrid, Spain), 1 × reaction buffer (750 mM Tris-HCl (pH 9.0), 500 mM KCl, 200 mM (NH_4_)_2_SO_4_), 0.2 mM of each dNTP, 1.5 mM MgCl_2_ and 0.25 μM of each primer (Metabion, Martinsried, Germany). The primer sequences are presented in Table 2. PCR amplifications were conducted in DNA Engine thermal cycler (Bio-Rad Laboratories, Hercules, CA, USA). Thermal cycling conditions were as follows: initial denaturation step at 95 °C for 5 min, 32 cycles at 95 °C for 30 s, 30 s at 63 °C annealing temperature and 60 s at 72 °C for the *SENP1* gene polymorphism, and were as follows: initial denaturation step at 95 °C for 5 min, 30 cycles at 95 °C for 30 s, 30 s at 55 °C annealing temperature and 60 s at 72 °C for the *SENP2* gene polymorphism.

**Table 2:** Primer sequences of the c.1691 + 36C > T polymorphism of the *SENP1* gene (rs12297820) and c.902C > A polymorphism of the *SENP2* gene (rs6762208)

Gene/Polymorphism	Primer sequences
*SENP1* gene/c.1691 + 36C > T (rs12297820)	
Sense	5′-AAGTCTGGCAAAAGGTTCCA -3′
Antisense	5′-TTGCTCCCATTAGGGCATAC-3′
*SENP2* gene/c.902C > A (rs6762208)	
Sense	5′-CAGAAAAGTTCGTTGACTCCTG-3′
Antisense	5′-CATGGGCACCAAAACATAAG-3′

The products of the c.1691 + 36C > T and c.902C > A polymorphisms were digested overnight at 37 °C with 0.2 U of the restriction enzyme *Eco*RV and *Psh*AI (NEB New England Biolabs, Ipswich, MA, USA), respectively. The PCR products were separated into 8 % polyacrylamide gel, stained with ethidium bromide and viewed under UV light.

### Statistical Analysis

Statistical analysis was performed using STATISTICA 8.0 package (Statsoft, Tulsa, OK, USA). Distributions of genotypes and alleles between groups were tested using the χ^2^ analysis. The Hardy-Weinberg equilibrium was checked using the χ^2^ test to compare the observed genotype frequencies with the expected frequencies among the case and control subjects. For each SNP, the odds ratios (ORs) and 95 % confidence intervals (CIs) were calculated. A linkage between genotype, cancer and clinical parameters was assessed by the logistic regression.

## Results

### Genotype Analysis

**Fig. 1:**
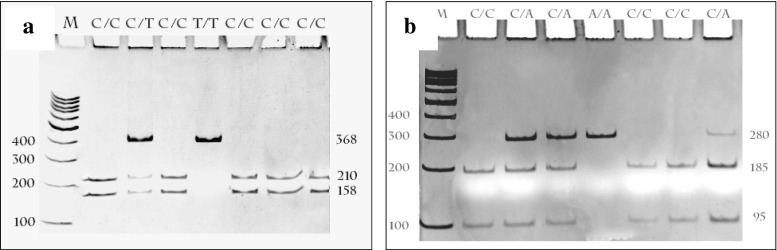
Genotypes of the of the c.1691 + 36C > T polymorphism of the *SENP1* gene (rs12297820) (**a**) and c.902C > A (p.Thr301Lys) polymorphism of the *SENP2* gene (rs6762208) (**b**) analyzed by 8 % polyacrylamide gel electrophoresis stained with ethidium bromide and viewed under UV light. Lane M shows GeneRuler TM 100 bp molecular weight marker

Breast cancer patients and controls were divided into groups corresponding to three genotypes. The distribution of genotypes of polymorphic variants of the *SENP1* and *SENP2* genes for cancer patients and controls is shown in Table 3. The genotype distributions for the c.1691 + 36C > T of the *SENP1* gene and c.902C > A of the gene *SENP2* were not in agreement with those predicted by the Hardy-Weinberg equilibrium (*p* < 0.05), except for that of the c.1691 + 36C > T polymorphism of the *SENP1* gene for the patients group. In the case of the *SENP1* gene polymorphism we did not find any association between this polymorphism and breast cancer risk. In the case of *SENP2* gene polymorphism we observed higher risk of breast cancer for carriers of the A allele (OR =1.33; 95 % CI 1.04–1.69). Our analysis also showed the genotype C/C (OR =0.67, 95 % CI 0.48–0.93) and the allele C (OR =0.75, 95 % CI 0.59–0.69) of this polymorphism decrease a risk of breast cancer. Figure 1 presents a representative gels from analysis of the c.1691 + 36C > T polymorphism of the *SENP1* gene (A) and the c.902C > A polymorphism of the *SENP2* gene (B).

**Table 3:** The genotype and allele frequency and odds ratios (OR) of the c.1691+ 36C > T polymorphism of the *SENP1* gene (rs12297820) and the c.902C > A polymorphism of the *SENP2* gene (rs6762208) in breast cancer patients and controls

Genotype or allele	Breast cancer patients (*n* = 324)		Controls (*n* = 335)		OR (95 % Cl)	*p*
	Number	Frequency	Number	Frequency		
*SENP1* gene/c.1691 + 36C > T (rs12297820)						
C/C	253	0.78	257	0.77	1.08 (0.75–1.56)	0.674
C/T	65	0.20	67	0.20	1.00 (0.69–1.47)	0.984
T/T	6	0.02	11	0.03	0.56 (0.20–1.52)	0.253
C	571	0.88	581	0.87	1.12 (0.82–1.54)	0.463
T	77	0.12	89	0.13	0.89 (0.65–1.22)	0.463
*SENP2* gene/c.902C > A (rs6762208)						
C/C	82	0.25	113	0.34	**0.67** **(0.48–0.93) ↓**	0.018
C/A	191	0.59	180	0.54	1.24 (0.91–1.68)	0.177
A/A	51	0.16	42	0.12	1.30 (0.84–2.02)	0.239
C	355	0.55	406	0.61	**0.75 (0.59–0.96) ↓**	0.021
A	293	0.45	264	0.39	**1.33 (1.04–1.69) ↑**	0.021

Data in boldface are statistically significant

### Clinical Parameters of Breast Cancer Patients and *SENP1* and *SENP2* Genes Polymorphism

We checked the distribution of genotypes and frequency of alleles of the *SENP1* and *SENP2* genes polymorphisms in groups of patients with different hormone receptor status, patients with positive and negative lymph node status and patients with different tumor grade. Odds ratio analysis showed a higher risk of metastases in women with the genotype C/C (OR =2.07, 95 % CI 1.06–4.05) and allele C (OR =2.10 95 % CI 1.10–4.01) of the c.1691 + 36C > T *SENP1* gene polymorphism (Table 4). Moreover, we observed reduced risk of metastases in women with the allele T (OR =0.48, 95 % CI 0.25–0.91) in this polymorphic site. In the case of *SENP2* gene polymorphism we observed that the A/A genotype correlated with the lack of estrogen receptor (OR =1.94, 95 % CI 1.04–3.62) (Table 5).

**Table 4:** The genotype and allele frequency and odds ratios (OR) of the c.1691 + 36C > T polymorphism of the *SENP1* gene (rs12297820) in groups of patients with breast cancer with positive and negative lymph node status

Genotype or allele	Node positive (*n* = 102)		Node negative (*n* = 133)		OR (95 % Cl)	*p*
	Number	Frequency	Number	Frequency		
C/C	87	0.85	98	0.737	**2.07 (1.06–4.05)↑**	0033
C/T	15	0.15	33	0.248	0.52 (0.27–1.03)	0059
T/T	0	0	2	0.015	-	0.992
C	189	0.93	229	0.86	**2.10 (1.10–4.01)↑**	0.024
T	15	0.07	37	0.14	**0.48 (0.25–0.91)↓**	0.024

Data in boldface are statistically significant

**Table 5:** The genotype and allele distribution and odds ratios (OR) of the c.902C > A polymorphism of the *SENP2* gene (rs6762208) in groups of patients with different estrogen receptor (ER) status

Genotype or allele	ER negative (*n* = 88)		ER positive (*n* = 235)		OR (95 % Cl)	*p*
	Number	Frequency	Number	Frequency		
C/C	19	0.21	63	0.27	0.75 (0.42–1.35)	0.338
C/A	49	0.56	141	0.60	0.84 (0.51–1.37)	0.483
A/A	20	0.23	31	0.13	**1.94 (1.04–3.62) ↑**	0.006
C	87	0.49	267	0.57	0.69 (0.47–1.02)	0.064
A	89	0.51	203	0.43	1.45 (0.98–2.14)	0.014

Data in boldface are statistically significant

We did not observe any association between: progesterone receptor status, HER2 expression and tumor grade described by Bloom-Richardson grading system, and the distribution of genotypes and frequency of alleles for these polymorphisms of the *SENP1* and *SENP2* genes (data not shown).

## Discussion

In the present study we correlated the genetic constitution of breast cancer patients expressed by polymorphic variants of two SUMO-specific cysteine proteases − SENP1 and SENP2, with clinical parameters of patients, including lymph node status, tumor grade, hormone receptors (estrogen and progesterone receptors) and epidermal growth factor receptor 2 (HER2) expressions. The c.1691 + 36C > T gene polymorphism (rs12297820) is located in the intron region of *SENP1* gene. We did not observe any correlation between this polymorphism and breast cancer risk (Table 3). However, we found a higher risk of metastases in women with the genotype C/C (OR =2.07, 95 % CI 1.06–4.05) and allele C (OR =2.10 95 % CI 1.10–4.01) in this polymorphic site (Table 4.). Moreover, we observed reduced risk of metastases in women with the allele T (OR =0.48, 95 % CI 0.25–0.91) (Table 4.). Our study for the first time showed a link between the variability of *SENP1* gene and metastasis in breast cancer. SENP1 was shown to have a pro-oncogenic role in many types of cancer. Clinical data showed that SENP1 was positively associated with lymph node metastasis and TNM stage pancreatic ductal adenocarcinoma (PDAC) [12]. Furthermore, knockdown of *SENP1* by SENP1-siRNA inhibited pancreatic cancer cell proliferation, migration, and invasion, suggesting that SENP1 played an important role in PDAC progression and metastasis. Silencing of *SENP1* results in down regulation of MMP-9, which is pivotal for PDAC cells growth and migration [12]. SENP1 can also transform normal prostate epithelia to a dysplastic state and directly modulate several oncogenic pathways in prostate cells, including AR, c-Jun, and Cyclin D1 [2, 10]. Assessment of tissue from human prostate cancer patients indicates elevated mRNA levels of SENP1 and the SUMO2/3 deconjugating enzyme, SENP3. The induction of SENP3 in cancer cells initiates the angiogenic pathway; specifically SENP3 regulates the transcriptional activity of hypoxia-inducible factor 1α (HIF1α) via desumoylation of the co-regulatory protein p300. Unlike prostate cancer, enhanced sumoylation is favored with onset of breast cancer and correlated with the reduced SENP6 mRNA levels found in several breast cancer tissue arrays. Preventing enhanced SUMO conjugation of cellular substrates in breast cancer cells reduces tumorigenesis [2].

Latest studies demonstrated that low expression of miR-145 was correlated with high expression of SENP1 in prostate cancer cell line PC-3 [11]. The transient introduction of miR-145 caused cell cycle arrest in PC-3 cells, and the opposite effect was observed when miR-145 inhibitor was transfected. Further studies revealed that the SENP1 3′-untranslated region was a regulative target of miR-145 in vitro. MicroRNA-145 also suppressed tumor formation in vivo in nude mice [11]. Silencing SENP1 level in highly metastatic prostate cancer cells perturbs their ability to metastasize to the bone and initiates secondary tumors. The expression of two critical bone remodeling proteins, matrix metalloproteinase 2 (MMP2) and MMP9, is regulated by SENP1 through the HIF1α signaling pathway. All these results show the contribution of SENP1 to the progression of prostate cancer, and suggest that SENP1 may be a prognostic marker and a therapeutic target for metastasis in prostate cancer patients [11, 13].

In the case of the *SENP2* gene polymorphism - c.902C > A, p.Thr301Lys (rs6762208), we observed correlation between this site and breast cancer risk (Table 3). Moreover, we noticed that the A/A genotype correlated with the lack of estrogen receptor (OR =1.94, 95 % CI 1.04–3.62) (Table 5.) Recently, it was found that SENP2 significantly repress estradiol-induced transcriptional activity in breast cancer cells (MCF-7 and T47D) [17]. It was also demonstrated that ERα repression by SENP2 is independent of its SUMO protease activity and requires a transcriptional repressive domain located in the amino-terminal end of the protease. This domain recruits the histone deacetylase, HDAC3 to be fully active. Furthermore, SENP2 robustly repressed estrogen-dependent and independent proliferation of MCF-7 cell, and this effect required both the proteolytic and transcriptional activities of SENP2. The data identifies SENP2 as a classical transcription co-regulatory [17].

Goeres et al. (2011) demonstrated that SENP2, although concentrated at the nuclear basket, is dynamically associated with nuclear pore complexes (NPCs) [18]. This association is mediated by multiple targeting elements within the N-terminus of SENP2 that function cooperatively to mediate NPC localization. Previously, it was also shown that SENP2 associates with the nuclear face of nuclear pores and that this association requires protein sequences near the N terminus of SENP2 [19]. The changes in pore organization and function observed after depletion of SENP1 and SENP2 indicate that these proteases contribute to nuclear transport, perhaps by controlling the configuration of nuclear pore complexes or modulating the interactions that can take place with pore components to promote particular types of traffic [20]. It is also possible that particular components of the soluble transport machinery (via their SUMO status) are functionally modulated by these enzymes, affecting the kinetics of transport for particular cargo. Overall the pore-associated SUMO proteases contribute to the fidelity of nuclear pore assembly and the robustness of nuclear import, suggesting an important means of contributing to cellular fitness [20].

SENP2 shuttles between the nucleus and the cytoplasm through an NLS (nuclear localization signal) identified at residues 29 to 49 and an NES (nuclear export signal) at residues 317 to 332 [4]. An autonomous NES localizes in the non-conserved central domain of the protein that functions through the CRM1 (Chromosome Region Maintenance 1/exportin1/Exp1/Xpo1)-dependent nuclear export pathway. Studied by us polymorphic site in *SENP2* gene - c.902C > A, p.Thr301Lys is located near the NES sequence. The observed at this point substitutions of amino acids may affect the activity and substrate specificity of SENP2 as well as nucleocytoplasmic traffic.

SUMO proteases could represent a group of new targets for therapeutic intervention in certain human diseases, including cancer, for which studies have suggested that altered sumoylation may contribute to both the onset and progression of the disease [4]. Consequently, in recent years, considerable progress has been made towards the identification of various SENP isoforms inhibitors such as protein-based, peptidyl and small molecule inhibitors [21]. Detailed studies are needed to clarify therapeutic potential these inhibitors.

## Conclusion

The variability of the *SENP1* and *SENP2* genes may play a role in breast cancer occurrence. Probably, the examined polymorphisms of the *SENP1* (c.1691 + 36C > T, rs12297820) and *SENP2* (c.902C > A, p.Thr301Lys, rs6762208) genes cannot be independent markers of breast cancer but our studies may be useful in building a set of molecular and clinical markers helpful for diagnosis and treatment of breast cancer.
